# Effect of UHT Thermal Treatment on the Secondary Structures of Milk Proteins: Insights From FTIR Analysis and Potential Allergenic Activity

**DOI:** 10.1155/2024/1880779

**Published:** 2024-08-22

**Authors:** Sergio-Miguel Acuña-Nelson, Leslie-Patricia Henríquez-González, Vieroska-Belén Sepúlveda-Villagra, Mauricio Opazo-Navarrete, Samuel Durán-Agüero, Julio-Enrique Parra-Flores

**Affiliations:** ^1^ Food Engineering Department University of Bío-Bío, Chillán 3780000, Chile; ^2^ Agriaquaculture Nutritional Genomic Center (CGNA), Temuco 4780000, Chile; ^3^ Nutrition and Dietetic School San Sebastián University, Providencia 7500000, Chile; ^4^ Nutrition and Public Health Department University of Bío-Bío, Chillán 3780000, Chile

**Keywords:** allergenic activity, casein, FTIR

## Abstract

Although thermal treatments are beneficial for the preservation and safety of milk, they can also alter its immunogenic activity by affecting its protein components. To achieve precise results, it is essential to identify the specific proteins that cause food allergies. Therefore, investigating the possible alterations of cow's milk proteins (CMPs) resulting from thermal treatments is necessary. In this study, the Fourier transform infrared spectroscopy (FTIR) technique was used to analyze the effect of UHT thermal treatment on the secondary structures of milk casein. Using the second derivative, six characteristic peaks were identified in the Amide I region, ranging from 1700 to 1600 cm^−1^. It was found that thermal treatments produce shifts in absorption peaks, indicating changes in protein conformation and possibly in allergenic activity. These shifts were clearly identified in the first characteristic peak of samples M8 and M9, from 1621 to 1600 cm^−1^. The results suggest that thermal treatments may promote protein aggregation by increasing *β* turns and reducing *β* sheets and *α* helices, which could enhance the allergenic potential of the proteins and facilitate the formation of complexes between different milk proteins, such as *β*-lactoglobulin and *κ*-casein. Further studies are needed to experimentally validate the allergenic activity of proteins modified by thermal treatments, as only an analytical method (FTIR) was used to evaluate the secondary structures of the proteins.

## 1. Introduction

Milk is an important food in the diet as it is a great source of vitamins and minerals (calcium, vitamin A, and vitamin B6) that aid in the growth and development of infants, as well as strengthen and/or improve bones, hair, teeth, and skin [1, 2]. The consumption of this food is one of the first introduced in the diets of babies and children, as it is rich in proteins, bioactive components, and various immunoactive substances [3]. Although there are similarities between cow's milk and human milk, there are differences in terms of protein concentration and type that cause an immune response in people [4].

However, consumption of cow's milk proteins (CMPs) carries an inherent risk for developing cow's milk allergy (CMA). CMA is one of the most prevalent food allergies in infants and young children, affecting 0.5%–3% [5]. Components of cow's milk can cause intolerances or allergies, which due to their symptoms are often confused and thought to be the same, which is why it is necessary to clarify these two types of reactions. Intolerance is a nonimmune reaction that affects humans due to a specific component of milk, lactose, causing disorders in digestion, absorption, or metabolism [2]. Whereas CMA occurs when CMPs cause an immuno-mediated response that occurs consistently with ingestion. These responses can be classified as immunoglobulin E (IgE) mediated, non-IgE mediated, and mixed (IgE combined with non-IgE) [6]. Additionally, they are measured through IgE, generating adverse reactions to these proteins that can occur after ingestion by sensitized/allergic individuals [2]. Likewise, non-IgE and IgE-mediated food allergies have been identified and characterized as the most relevant allergens in cow's milk to the whey fraction, lactoglobulins, and especially to casein [3].

For common people, CMPs are harmless substances. For people allergic to CMP, they are largely sensitive, which consequently produces characteristic inflammation of allergies. Unprocessed cow's milk has a concentration of proteins three times higher than human milk. Cow's milk contains 20% whey proteins, *β*-lactoglobulin, and *α*-lactalbumin, and 80% proteins such as casein, *α*-(S1 and S2), *β*-, and *κ*-caseins, where the latter and *β*-lactoglobulin are the most frequent to cause allergic reactions in allergic individuals [4, 7, 8].

Whey proteins represent 20% of CMP. It is of great importance to the food industry, since the concentrate of this protein is mainly used as a substitution for fat, due to its high emulsifying capacity to bind water and fat, stabilizing and gel-forming properties [8–10]. These proteins are thermolabile, which generates changes in their allergenicity [11]. When *β*-lactoglobulin is subjected to temperatures between 50°C and 90°C, it shows high antigenicity and allergenicity, while being subjected to temperatures higher than 90°C, *β*-lactoglobulin decreases its allergenicity due to the exchange of sulfhydryl-disulfide, which creates an improvement in changes after the destruction of epitopes formed on the surface of the molecule [12–15].

On the other hand, casein does not maintain a structural order; however, secondary and tertiary structures such as *β*-caseins and *κ*-caseins are presented. Since casein does not maintain a compact and flexible structure, it is called poorly immunogenic [16].

Due to milk's richness in nutrients, high water activity, and neutral pH, it provides an ideal environment for the growth and proliferation of various microorganisms, such as *Pseudomonas*, *Lactococcus*, *Acinetobacter*, *Enterobacteriaceae*, *Bacillus cereus*, *Bacillus* spp., *Bacillus lonarensis*, *Bacillus thuringiensis*, *Bacillus licheniformis*, *Clostridium* spp., *Salmonella* spp., and *Staphylococcus aureus* [17]. Therefore, is necessary to apply a thermal treatment to inactivate microorganisms and increase the shelf life of the food [4, 18].

In the dairy industry, in addition to pasteurization at 71°C for 15–40 s, we find sterilization at 110°C–120°C for 10–20 min and ultrahigh temperature (UHT) at 135°C–145°C for 0.5–4 s (UHT), with the latter being one of the most widely used today [11]. Although the objective of these treatments is to decrease microbial pathogens in raw milk, these treatments alter the molecular structure of proteins, and this structural alteration presents as allergens for the immune system [4, 19].

For centuries, milk has been consumed and various thermal treatments are currently used that undoubtedly favor the preservation and safety of the product for the consumer. However, although these treatments offer benefits, they could also affect the protein components of milk and hence alter the general immunogenic activity of milk. Knowing the specific proteins/peptides that cause food allergies is one more step towards obtaining precise results through new and more efficient diagnostic tools, such as microarrays. Microarrays determine different patterns of epitope binding, which allows the differentiation of clinical phenotypes of CMA, but their cost is high [20, 21]. Therefore, it is essential to investigate the possible alterations of CMPs due to thermal treatments with fast and low-cost techniques. In this study, we used Fourier transform infrared spectroscopy (FTIR) to determine the possible changes produced in the protein casein (the most abundant and least thermolabile) of commercial milk subjected to UHT thermal treatment and compared the results with those of pure casein to better understand how thermal treatments, specifically the UHT process, affect CMPs, particularly casein. This would be important not only for gaining a better understanding of the effects of production processes on the nutritional and functional quality of dairy products but also for improving milk processing and preservation methods to minimize negative impacts.

## 2. Materials and Methods

In this study, casein is used as a control since it provides a more stable and consistent control in terms of composition and protein content compared to raw milk, which could have also been used as a control. Raw milk can vary in its protein content due to factors such as cow breed, diet, and other environmental factors. Therefore, using casein as a control allows us to establish a more constant and controlled reference for analyzing the changes induced by thermal treatment. On the other hand, the selected milk samples were from various brands of UHT milk of Chilean origin, marketed in Chile. Nine commercial brands of 1 L whole milk in tetra pack format were used, which were purchased locally. All milks studied were from the same country of origin, and all mentioned, as an ingredient on their labels, natural fluid milk subjected to UHT processing. According to the labels of the milk samples, all of them presented the same composition: 31 g/L of fat, 31 g/L of protein, and 46 g/L of carbohydrates. Milks were received and labeled using the symbols M1 to M9.

### 2.1. Casein Control

A 2.5% (w/v) solution of bovine milk casein (Sigma-Aldrich, C7078) was used as a control sample. Casein was dissolved in distilled water [22], allowed to rest for 12 h at refrigeration temperature, and then placed on a magnetic stirrer for 30 min until completely dissolved.

### 2.2. FTIR Analysis

Analysis of the casein control and milk samples was performed using a FTIR instrument (Shimadzu, Prestige21) equipped with a universal ATR sampling accessory (PIKE, MIRacle™). Distilled water was used as background. For each sample, scans were performed from 600 to 4000 cm^−1^, with a step of 4 cm^−1^, for a total of 128 scans per sample. Each sample was analyzed in triplicate, and the results were averaged using custom routines. Measurements were taken at room temperature using approximately 0.1 ml, approximately, of each sample, which were placed onto the surface of the ATR crystal. The Amide I region (1700 cm^−1^ and 1600 cm^−1^) was specifically manipulated due to the high signal-to-noise ratio in this region, as described by Haque et al. [23]. Resulting protein spectra were smoothed using a 9-point Savitsky-Golay function, and normalized second-derivative spectra were obtained using IRsolution software version 1.10 (Creon Lab Control AG, Shimadzu Corporation Pte. Ltd., Kyoto, Japan) [24].

## 3. Results and Discussions

### 3.1. FTIR Spectra

The use of water as a background in FTIR is not common; however, in specific cases, it is possible to employ distilled water as a background in the FTIR technique for analyzing casein proteins in milk. While it is true that milk has a buffered pH and calcium is crucial for the formation of casein micelles, there are situations where this choice is justified in FTIR studies. Some investigations focus on analyzing the secondary structure characteristics of casein proteins, such as changes in absorption bands that indicate modifications in protein conformation [25–27]. In our case, our objective was to evaluate the changes induced by thermal treatment in the secondary structure of casein proteins, and the use of distilled water as a background allows for obtaining a pure spectrum of the proteins under analysis. This facilitates the precise detection of specific changes in absorption bands related to protein conformation.

Furthermore, analyzing whole milk without separating the proteins from other components allows for a more comprehensive and realistic representation of the system in its natural state. Milk is a complex matrix with a variety of components that interact with each other, and the properties and changes in the casein proteins may be influenced by the presence of lipids, carbohydrates, salts, and whey proteins. By keeping the system intact, the interaction and potential effect of all these components on the casein proteins can be better captured.

By measuring the FTIR spectra of whole milk, results can be obtained more quickly and easily, which can be a relevant consideration in a preliminary or exploratory study, such as the one conducted in this research. On the other hand, we must consider that milk is consumed and processed in its complete form, and the thermal treatment will affect the casein proteins in the context of milk as a whole. By measuring the FTIR spectra of whole milk, directly applicable information to the system found in the market and consumer is obtained, which may have practical implications for the dairy industry and the quality of dairy products.

**Figure 1:**
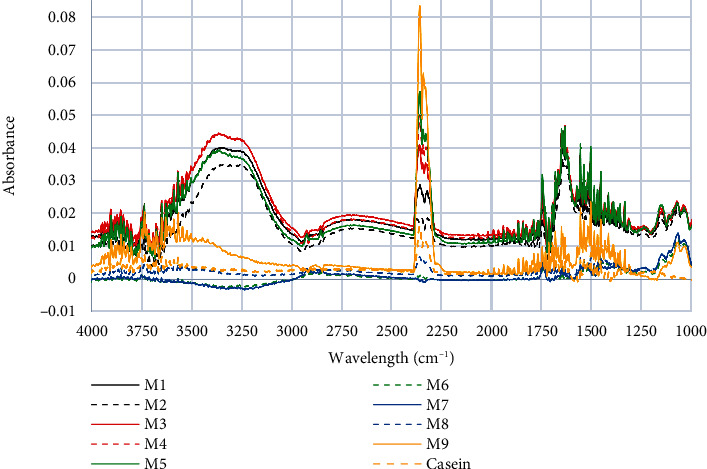
General FTIR spectra of milks and casein control.

Figure 1 shows the total spectrum of the samples, in which relevant peaks of Amides I, II, and A can be observed. The peak A, at approximately 3300 cm^−1^, is one of the most prominent peaks and an indicator of milk protein quality [28]. This peak represents the NH stretching vibration mainly associated with whey protein due to the amino acid content that this band represents [29].

**Figure 2:**
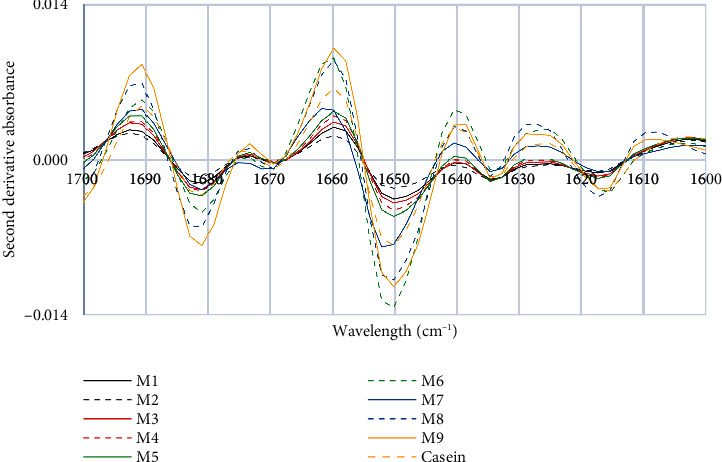
Characteristic peaks of the second derivative of the FTIR spectra of milk and casein control samples.

Figure 2 depicts distinct curves of milk samples that were obtained using a specific wavelength range of 1600–1700 cm^−1^, corresponding to the Amide I band [30]. These spectra were collected at a resolution of 4 cm^−1^ and were further processed by applying a 9-point smoothing filter, normalization, and second-derivative analysis. When observing Figure 2, peaks of varying intensity can be identified, which are associated with the essential vibration modes of each molecular bond present in the analyzed samples. This IR spectrum results from the absorption of wavelengths associated with a specific group of chemical bonds within the molecule [31], and the peak height reflects the concentration of a particular molecule [32, 33]. Amide I absorption depends on the structure of the protein backbone. The main band maintains a position that shifts towards longer wavelengths (down in the spectrum) as the helix length increases, as well as when the helix folds into coiled spirals and when the helix is subject to solvent (maximum absorption at 1640–1650 cm^−1^ in H_2_O, at 1629–1640 cm^−1^ in 2H_2_O) [34]. In the Amide I region, Cepero-Betancourt et al. [24] assigned the bands from 1609 cm^−1^ to 1628 cm^−1^, 1628 cm^−1^ to 1639 cm^−1^, and 1675 cm^−1^ to 1695 cm^−1^ to intermolecular, intramolecular, and antiparallel *β*-sheet structures, respectively. In addition, the random coil was assigned to 1640 cm^−1^ to 1650 cm^−1^, the 310-helix was assigned to 1655 cm^−1^ to 1665 cm^−1^, and the *β*-turn conformation was assigned to the peaks between 1675 cm^−1^ and 1685 cm^−1^.

In Figure 2, the effect of UHT treatment on the change in secondary structures can be observed. Subjecting milk to UHT results in a significant alteration in its structure, as indicated by the analysis of its most prominent peaks. Specifically, the peaks corresponding to beta sheets are less concentrated compared to those of beta turns. Zhu et al. [35] conducted studies on ultrahigh pressure (UHP) combined with higher temperature treatments, and the results of the study showed that this technique has a significant effect on the protein structure, which could reduce its allergenicity and increase the safety of beef, milk, and beef products. In particular, a decrease in the number of alpha helices and an increase in beta sheets, beta turns, and random coils have been observed in the BSA protein. The beta-turn structure is a folded conformation of proteins, while the antiparallel beta sheet is formed when protein molecules aggregate. Thus, a modification of these structures may result in the alteration or destruction of antigenic epitopes, which are regions of proteins that interact with antibodies and trigger an immune response, impacting the ability of proteins to interact with the immune system and, therefore, their allergenicity.

**Figure 3:**
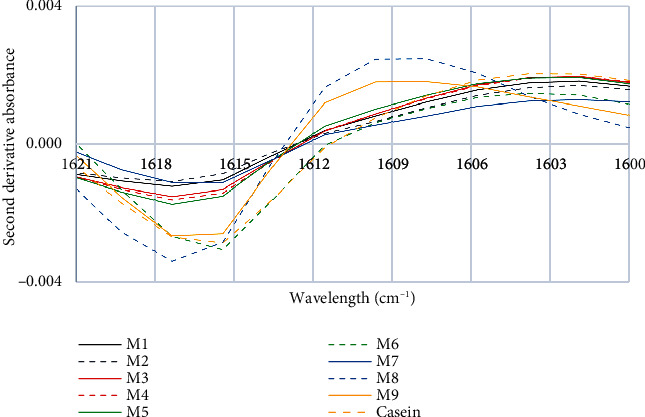
First characteristic peak of the second derivative of the FTIR spectra of milk and casein control samples.

In Figure 3, the samples of commercial milk brands M8 and M9 show a significant peak shift towards higher wavelengths, with ~1603 cm^−1^ being the peak of the control sample and ~1609 cm^−1^ being the peak of the aforementioned milks. The peak at 1603 cm^−1^ reflects an intense band due to the N-H bending mode of the NH_2_ group [36]. According to Daniloski et al. [37], the range between 1614 cm^−1^ and 1601 cm^−1^ corresponds to protein side chains. Eissa et al. [38] mention that a small shoulder is formed at 1605 cm^−1^, which reflects the residues of the side chain, and it is most likely that this comes from proline, due to its high presence in casein, and the effect it has on it, which is to inhibit the formation of ordered helical structures, while the presence of *κ*-casein organizes some regions into short *α*-helical structures and *β*-laminar fragments [39], and they establish that these side chain residues can be composed of 8 amino acids. Thus, although *κ*-casein is resistant to denaturation at high temperatures due to its amino acid sequence, the presence of these side chains could be responsible for causing allergies in people who consume milk. Any changes in this peak could suggest that we are dealing with more allergenic compounds.

**Figure 4:**
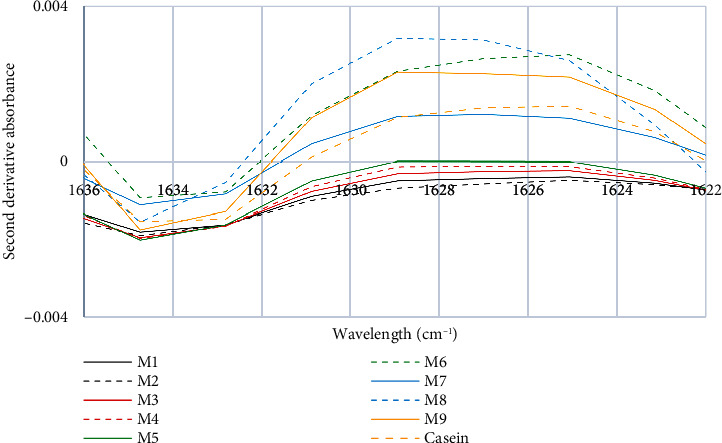
Second characteristic peak of the second derivative of the FTIR spectra of milk and casein control samples.

In Figure 4, it can be observed that the commercial milk brands M7, M8, and M9 exhibit an absorption peak at 1629 cm^−1^, while the control sample does so at 1625 cm^−1^. This peak corresponds to the *β*-sheet intramolecular structure of proteins, according to Daniloski et al. [37]. These milks also show a higher intensity of the peak than the rest of the commercial milks, which have wider and less concentrated curves. The angular bending vibration bands of the carbon-nitrogen bond of Amide I, recorded at 1625 cm^−1^ and 1630 cm^−1^, are indicative of the protein's secondary structure. In particular, the band at 1630 cm^−1^ is associated with parallel *β*-sheets, while the band at 1625 cm^−1^ may vary slightly due to molecular interactions and sample conformation. In some cases, the band at 1625 cm^−1^ has been related to the presence of *α*-helix secondary structures. Calculations performed support these results [40] and allow us to conclude that parallel *β*-sheets show their initial absorption at the peak at 1630 cm^−1^ [41]. On the other hand, it has been demonstrated that short *α*-helices with a residue value less than 6 do not always exhibit the characteristic absorption of the *α*-helix but may present a variety of bands in the Amide I region [34]. Proline residues are fundamental in creating *β*-turns in caseins, thanks to their cross-linking capacity. In the case of bovine *β*-casein, 35 proline residues have been identified and distributed along the polypeptide chain, which favors the formation of PPII structures [42, 43]. According to these authors, the sensitivity of *β*-turn structures to temperature is more relevant than their sensitivity to pH in terms of allergenicity. This coincides with the finding that an increase in temperature can cause the conversion of *β*-turns and *β*-sheets to PPII conformations, which can have implications for the stability and potential allergenicity of proteins.

**Figure 5:**
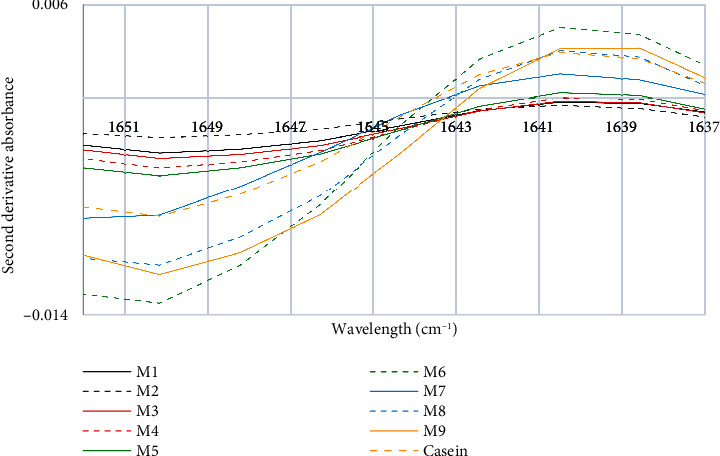
Third characteristic peak of the second derivative of the FTIR spectra of milk and casein control samples.

In Figure 5, it can be observed that the control sample maintains a peak at 1640 cm^−1^, which corresponds to the random coil structure [37], with a range between 1645 cm^−1^ and 1638 cm^−1^. On the other hand, in the M9 and M6 samples, the peak at 1640 cm^−1^ is more concentrated, which could indicate the presence of *β*-casein and the thermal treatments applied in pasteurization. In a study on *β*-casein with ferulic acid, a main peak was observed in the Amide I region at 1639 cm^−1^ in the heat-treated sample [44]. Heating of milk has been associated with an increase in the intensity of intramolecular structures of *β*-sheet, random coil, and *α*-helix. Additionally, it has been determined that a peak in the Amide I region at 1640 cm^−1^ is associated with the vibration of the carbonyl group, and this intense vibration can promote the formation of irregular structures and *β*-helices in *β*-casein [37, 45, 46]. These structural modifications due to the application of heat can result in irregular structures and impact the physical and chemical properties of *β*-casein.

**Figure 6:**
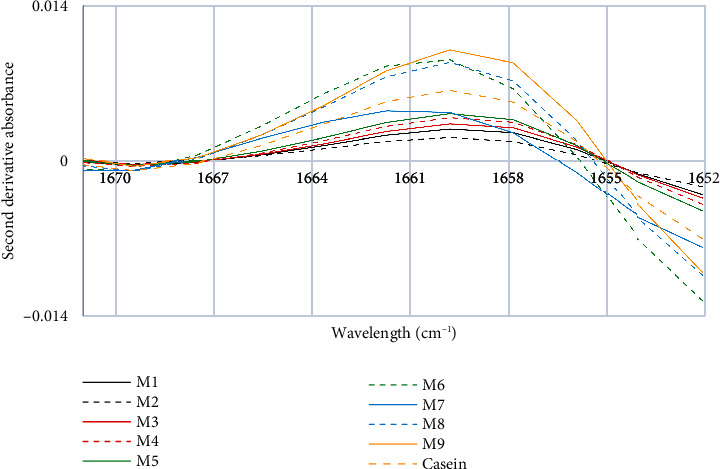
Fourth characteristic peak of the second derivative of the FTIR spectra of milk and casein control samples.

In Figure 6, it can be observed that commercial milk samples, except for the M7 brand, show a shift towards higher wavelengths, 1662 cm^−1^, in the Amide I spectrum. The fourth characteristic peak, which has the highest absorbance in the Amide I spectrum, shows higher concentrations in some milk samples than in the control sample, presenting a peak at 1659 cm^−1^. The wavelength ranges between 1664 cm^−1^ and 1646 cm^−1^ correspond to *α*-helix [37]. The Amide I group is located at a wavelength of 1653 cm^−1^ in the spectrum and is crucial for protein identification, as it is associated with the C=O bond of the amide and contributes 80% to the shape of the peak in the spectrum. Additionally, the peak indicates a stretching vibration that is also useful for protein identification and characterization.

**Figure 7:**
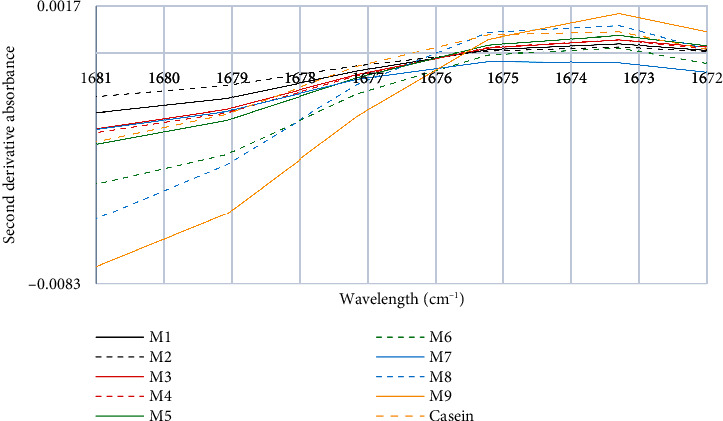
Fifth characteristic peak of the second derivative of the FTIR spectra of milk and casein control samples.

Figure 7 shows the fifth characteristic peak, whose peak consists of 1675 and 1673 cm^−1^. Previous studies have revealed that absorption bands between 1681 cm^−1^ and 1665 cm^−1^ are indicative of *β*-turns [37], and the absorption bands in the range of 1630 cm^−1^ to 1680 cm^−1^ are associated with the stretching of the C=O bond and the bending vibration of proteins, potentially due to their interaction with fat globules. Our results indicate that there is a similar relationship among the curves of absorption bands in the spectra. This finding is consistent with the fact that all milk samples studied contained the same amount of fat, which meets the requirement established by Chilean regulations to be called “whole milk”, which is 31 g of fat/liter.

**Figure 8:**
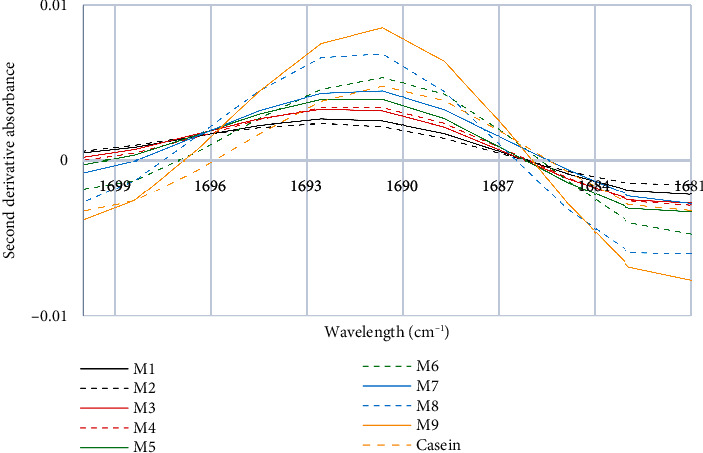
Sixth characteristic peak of the second derivative of the FTIR spectra of milk and casein control samples.

In Figure 8, a peak of absorption at 1690 cm^−1^ is observed, which is due to the stretching of the C=O bond in the carboxyl group of asparagine. This group is part of the glutamic acid and aspartic acid residues of *κ*-casein and the intensity of this peak reflects the amount and quality of *κ*-casein in the samples [45]. Heating alters the conformation of proteins and modifies their secondary structure, especially in the range of 1700 cm^−1^ to 1682 cm^−1^, where intermolecular or aggregated *β*-sheets of proteins are detected and these changes also depend on the milk solids content [46]. Our results do not show significant shifts or differences for the sixth characteristic peak. However, based on the obtained spectra, it can be seen that Samples M8 and M9 may have a higher concentration of *κ*-casein, which could be due to factors related to the origin of the milk.

## 4. Future Considerations

In the future, further studies are recommended to gain a more comprehensive understanding of casein proteins in UHT-processed milk. While the study focused on casein proteins, investigating whey proteins and the effects on casein micelles would also be valuable. Additionally, conducting separate analyses of casein proteins in future studies would contribute to a more precise understanding of the impacts of thermal treatment on these proteins.

Experimental studies have shown that UHT gradually increases the size of casein micelles. The relationship between casein micelles and the level of denatured whey proteins results in an increase in micelle size [47]. Additionally, recent studies have demonstrated that the application of thermal treatments, such as UHT, reduces the amount of phospholipids in milk [48]. This undoubtedly significantly affects micelle formation and interaction with milk proteins.

In addition to the above, further studies and more representative results are required. In future research, it is necessary to increase the sample size used in the study. This would involve analyzing a greater number of UHT-processed milk samples, including different commercial brands, types of milk (skimmed, semiskimmed, and lactose-free), and batches to capture inherent variability. Additionally, instead of relying solely on the FTIR technique, complementary analytical techniques can be employed to gain a more comprehensive understanding and validate the findings. For instance, high-performance liquid chromatography (HPLC) can be used to identify and quantify different proteins present in the milk. Moreover, biological assays can be conducted to assess the allergenic activity of proteins modified by thermal treatment. This may involve in vitro assays or experiments using animal models to evaluate the allergic response induced by heat-treated proteins. Furthermore, comparative studies between UHT-processed milk and raw unpasteurized milk could be conducted. This would enable the evaluation of how protein characteristics and allergenic properties vary in each type of milk and determine if thermal treatment has a significant impact. These studies would address the limitations of representativeness in the current study and yield more robust and generalizable results. This would contribute to a better understanding of the effects of thermal treatment on milk proteins and their potential implications for the allergenicity and quality of processed dairy products.

## 5. Conclusions

In this study, the FTIR technique was used to analyze the effect of UHT thermal treatment on the secondary structures of milk proteins, especially casein and whey proteins, which can cause food allergies. It was found that thermal treatments produce shifts in absorption peaks, indicating changes in protein conformation and possibly allergenic activity. These shifts were clearly identified for Samples M8 and M9 in the first (1621 to 1600 cm^−1^), second (1622 to 1636 cm^−1^), fourth (1652 to 1671 cm^−1^), and sixth (1681 to 1700 cm^−1^) characteristic peaks. Our results suggest that thermal treatments may promote protein aggregation by increasing *β*-turns and decreasing *β*-sheets and *α*-helices. This aggregation could increase the allergenic potential of proteins and facilitate the formation of complexes between *β*-lactoglobulin and *κ*-casein. However, further studies are required as only one analytical method (FTIR) was used to evaluate the secondary structures of proteins and an experimental validation of the allergenic activity of proteins modified by thermal treatments is lacking.

Furthermore, our findings provide a deeper understanding of how thermal treatments, such as UHT, can impact the structure of milk proteins and, consequently, their ability to trigger allergic reactions. This may lead to better identification of factors contributing to milk allergenicity and the formulation of strategies to minimize risks associated with the consumption of thermally processed dairy products.

Furthermore, for milk producers, understanding how thermal treatments affect the properties of milk proteins can influence decisions regarding the processing methods employed and the optimization of processes to ensure the quality and safety of dairy products. Additionally, it may foster innovation in the development of dairy products with improved allergenic profiles or alternatives for individuals with food allergies.

## Data Availability

Data is available on request.

## References

[1] Do A. B., Williams K., Toomer O. T. (2016). In vitro digestibility and immunoreactivity of bovine milk proteins. *Food Chemistry*.

[2] Villa C., Costa J., Oliveira M., Mafra I. (2018). Bovine milk allergens: a comprehensive review. *Comprehensive Reviews in Food Science and Food Safety*.

[3] Geiselhart S., Podzhilkova A., Hoffmann-Sommergruber K. (2021). Cow’s milk processing-friend or foe in food allergy?. *Food*.

[4] Jaiswal L., Worku M. (2022). Recent perspective on cow’s milk allergy and dairy nutrition. *Critical Reviews in Food Science and Nutrition*.

[5] Bøgh K. L., Nielsen D. M., Mohammad-Beigi H. (2024). Degree of hydrolysis is a poor predictor of the sensitizing capacity of whey- and casein-based hydrolysates in a Brown Norway rat model of cow’s milk allergy. *Food Research International*.

[6] Flom J., Sicherer S. H. (2019). Epidemiology of Cow’s Milk Allergy. *Nutrients*.

[7] Hochwallner H., Schulmeister U., Swoboda I., Spitzauer S., Valenta R. (2014). Cow’s milk allergy: From allergens to new forms of diagnosis, therapy and prevention. *Methods*.

[8] Ramachandran B., Yang C. T., Downs M. L. (2020). Parallel reaction monitoring mass spectrometry method for detection of both casein and whey milk allergens from a baked food matrix. *Journal of Proteome Research*.

[9] Hong L., Pan M., Xie X. (2021). Aptamer-Based Fluorescent Biosensor for the Rapid and Sensitive Detection of Allergens in Food Matrices. *Foods*.

[10] Khan M. U., Lin H., Ahmed I. (2021). Whey allergens: influence of nonthermal processing treatments and their detection methods. *Comprehensive Reviews in Food Science and Food Safety*.

[11] Verhoeckx K. C., Vissers Y. M., Baumert J. L. (2015). Food processing and allergenicity. *Food and Chemical Toxicology*.

[12] Kleber N., Hinrichs J. (2007). Antigenic response of *β*-lactoglobulin in thermally treated bovine skim milk and sweet whey. *Milchwissenschaft*.

[13] Bu G., Lu J., Zheng Z., Luo Y. (2009). Influence of Maillard reaction conditions on the antigenicity of bovine *α*-lactalbumin using response surface methodology. *Journal of the Science of Food and Agriculture*.

[14] Bloom K. A., Huang F. R., Bencharitiwong R. (2014). Effect of heat treatment on milk and egg proteins allergenicity. *Pediatric Allergy and Immunology*.

[15] Xu Q., Shi J., Yao M., Jiang M., Luo Y. (2016). Effects of heat treatment on the antigenicity of four milk proteins in milk protein concentrates. *Food and Agricultural Immunology*.

[16] Bhat M. Y., Dar T. A., Singh L. R., Gigli I. (2016). Casein proteins: structural and functional aspects. *Milk Proteins—From Structure to Biological Properties and Health Aspects*.

[17] Sun Y., Liu Y., Zhou W. (2024). Effects of ohmic heating with different voltages on the quality and microbial diversity of cow milk during thermal treatment and subsequent cold storage. *International Journal of Food Microbiology*.

[18] Abbring S., Hols G., Garssen J., van Esch B. C. A. M. (2019). Raw cow’s milk consumption and allergic diseases–The potential role of bioactive whey proteins. *European Journal of Pharmacology*.

[19] Braun-Fahrländer C., Von Mutius E. (2011). Can farm milk consumption prevent allergic diseases?. *Clinical & Experimental Allergy*.

[20] Ahrens B., Lopes de Oliveira L. C., Grabenhenrich L. (2012). Individual cow's milk allergens as prognostic markers for tolerance development?. *Clinical & Experimental Allergy*.

[21] Järvinen K. M., Sicherer S. H. (2012). Diagnostic oral food challenges: procedures and biomarkers. *Journal of Immunological Methods*.

[22] De Kruif C. G., Huppertz T., Urban V. S., Petukhov A. V. (2012). Casein micelles and their internal structure. *Advances in Colloid and Interface Science*.

[23] Haque M. A., Chen J., Aldred P., Adhikari B. (2015). Drying and denaturation characteristics of whey protein isolate in the presence of lactose and trehalose. *Food Chemistry*.

[24] Cepero-Betancourt Y., Opazo-Navarrete M., Janssen A. E. M., Tabilo-Munizaga G., Pérez-Won M. (2020). Effects of high hydrostatic pressure (HHP) on protein structure and digestibility of red abalone *(Haliotis rufescens*) muscle. *Innovative Food Science & Emerging Technologies*.

[25] Etzion Y., Linker R., Cogan U., Shmulevich I. (2004). Determination of protein concentration in raw milk by mid-infrared fourier transform infrared/attenuated total reflectance spectroscopy. *Journal of Dairy Science*.

[26] Hewavitharana A. K., van Brakel B. (1997). Fourier transform infrared spectrometric method for the rapid determination of casein in raw Milk. *Analyst*.

[27] Venyaminov S. Y., Kalnin N. N. (1990). Quantitative IR spectrophotometry of peptide compounds in water (H2O) solutions. II. Amide absorption bands of polypeptides and fibrous proteins in *α*-, *β*-, and random coil conformations. *Biopolymers*.

[28] Jayasooriya S. D., Torley P. J. (2011). Fourier transform infrared (FTIR) spectroscopy as a process analytical technology (PAT) tool for monitoring and understanding protein behaviour in dairy systems. *International Dairy Journal*.

[29] Wang T., Johnson W. (1994). Rapid determination of milk protein quantity and quality using Fourier transform infrared spectroscopy. *Journal of Dairy Science*.

[30] Wang J., Su Y., Jia F., Jin H. (2013). Characterization of casein hydrolysates derived from enzymatic hydrolysis. *Chemistry Central Journal*.

[31] Sierra I., González-Martin M. I., Corzo N., Carmona M. (2007). Chemical and microbiological characteristics of goat's milk produced in the Canary Islands. *International Dairy Journal*.

[32] Rodríguez-Otero J. L., Hermida M., Cepeda A. (1995). Determination of fat, protein, and total solids in cheese by near-infrared reflectance spectroscopy. *Journal of AOAC International*.

[33] Martinez M. M., Gomez C. A. (2013). Calidad composicional e higiénica de la leche cruda recibida en industrias lácteas de Sucre, Colombia. *Biotecnología en el Sector Agropecuario y Agroindustrial*.

[34] Barth A. (2007). Infrared spectroscopy of proteins. *Biochimica et Biophysica Acta (BBA)-Bioenergetics*.

[35] Zhu J., Garrigues L., Van den Toorn H., Stahl B., Heck A. J. (2018). Discovery and quantification of nonhuman proteins in human milk. *Journal of Proteome Research*.

[36] Gupta R., Sanotra S., Sheikh H. N., Kalsotra B. L. (2013). Room temperature aqueous phase synthesis and characterization of novel nano-sized coordination polymers composed of copper (II), nickel (II), and zinc (II) metal ions with p-phenylenediamine (PPD) as the bridging ligand. *Journal of Nanostructure in Chemistry*.

[37] Daniloski D., McCarthy N. A., Markoska T., Auldist M. J., Vasiljevic T. (2022). Conformational and physicochemical characteristics of bovine skim milk obtained from cows with different genetic variants of *β*-casein. *Food Hydrocolloids*.

[38] Eissa A. S., Puhl C., Kadla J. F., Khan S. A. (2006). Enzymatic cross-linking of *β*-lactoglobulin: conformational properties using FTIR spectroscopy. *Biomacromolecules*.

[39] Guevara-Garay L. A., Cuartas-Castaño D. A., Llano-Naranjo F. (2014). Kappa caseína de la leche: aspectos bioquímicos, moleculares, productivos y nutricionales. *Revista Médica de Risaralda*.

[40] Torii H., Tasumi M. (1992). Model calculations on the amide-I infrared bands of globular proteins. *The Journal of Chemical Physics*.

[41] Barth A., Zscherp C. (2002). What vibrations tell about proteins. *Quarterly Reviews of Biophysics*.

[42] Markoska T., Daniloski D., Vasiljevic T., Huppertz T. (2021). Structural changes of *β*-casein induced by temperature and pH Analysed by nuclear magnetic resonance, Fourier-transform infrared spectroscopy, and chemometrics. *Molecules*.

[43] Farrell H. M., Qi P. X., Wickham E. D., Unruh J. J. (2002). Secondary structural studies of bovine caseins: structure and temperature dependence of *β*-casein phosphopeptide (1-25) as analyzed by circular dichroism, FTIR spectroscopy, and analytical ultracentrifugation. *Journal of Protein Chemistry*.

[44] Condict L., Kaur J., Hung A., Ashton J., Kasapis S. (2019). Combined spectroscopic, molecular docking and quantum mechanics study of *β*-casein and ferulic acid interactions following UHT-like treatment. *Food Hydrocolloids*.

[45] Tarhan Ö., Kaya A. (2021). Investigation of the compositional and structural changes in the proteins of cow milk when processed to cheese. *LWT*.

[46] Gordillo-Delgado F., Bedoya-Pérez A. F., Delgado-Osorio H. D. (2020). Características mecánicas y térmicas de un poliuretano elaborado a partir de aceite de higuerilla (*Ricinus communis*) para la adhesión de elementos estructurales de guadua angustifolia kunth. *Revista UIS Ingenierías*.

[47] Syed Q. A., Hassan A., Sharif S. (2021). Structural and functional properties of milk proteins as affected by heating, high pressure, gamma and ultraviolet irradiation: a review. *International Journal of Food Properties*.

[48] Liu Y., Zhao J., Qiao W. (2024). Effect of infant formula production processes on phospholipid composition and structure of milk fat globules. *LWT*.

